# Liquid Chromatography-Mass Spectrometry-Based Rapid Secondary-Metabolite Profiling of Marine *Pseudoalteromonas* sp. M2

**DOI:** 10.3390/md14010024

**Published:** 2016-01-20

**Authors:** Woo Jung Kim, Young Ok Kim, Jin Hee Kim, Bo-Hye Nam, Dong-Gyun Kim, Cheul Min An, Jun Sik Lee, Pan Soo Kim, Hye Min Lee, Joa-Sup Oh, Jong Suk Lee

**Affiliations:** 1 Biocenter, Gyeonggi Institute of Science and Technology Promotion (GSTEP), Suwon, Gyeonggi-do 16229, Korea; wj0504@gstep.re.kr (W.J.K.); pan@gstep.re.kr (P.S.K.); hmlee@gstep.re.kr (H.M.L.); jsoh@gstep.re.kr (J.-S.O.); 2 Biotechnology Research Division, National Fisheries Research and Development Institute (NFRDI), Gijang-gun, Busan 46083, Korea; yobest12@korea.kr (Y.O.K.); nambohye@korea.kr (B.-H.N.); combikola@korea.kr (D.-G.K.); ancm@korea.kr (C.M.A.); 3 College of Herbal Bio-Industry, Daegu Haany University, Gyeongsan, Gyeongbuk 42158, Korea; jinheekim@dhu.ac.kr; 4 Department of Biology, Immunology Research Lab, BK21-plus Research Team for Bioactive Control Technology, College of Natural Sciences, Chosun University, Dong-gu, Gwangju 61452, Korea; junsiklee@chosun.ac.kr

**Keywords:** mass spectrometry, marine microbes, *Pseudoalteromonas*, secondary metabolite, quinolone alkaloid

## Abstract

The ocean is a rich resource of flora, fauna, and food. A wild-type bacterial strain showing confluent growth on marine agar with antibacterial activity was isolated from marine water, identified using 16S rDNA sequence analysis as *Pseudoalteromonas* sp., and designated as strain M2. This strain was found to produce various secondary metabolites including quinolone alkaloids. Using high-resolution mass spectrometry (MS) and nuclear magnetic resonance (NMR) analysis, we identified nine secondary metabolites of 4-hydroxy-2-alkylquinoline (pseudane-III, IV, V, VI, VII, VIII, IX, X, and XI). Additionally, this strain produced two novel, closely related compounds, 2-isopentylqunoline-4-one and 2-(2,3-dimetylbutyl)qunoline-4-(1H)-one, which have not been previously reported from marine bacteria. From the metabolites produced by *Pseudoalteromonas* sp. M2, 2-(2,3-dimethylbutyl)quinolin-4-one, pseudane-VI, and pseudane-VII inhibited melanin synthesis in Melan-A cells by 23.0%, 28.2%, and 42.7%, respectively, wherein pseudane-VII showed the highest inhibition at 8 µg/mL. The results of this study suggest that liquid chromatography (LC)-MS/MS-based metabolite screening effectively improves the efficiency of novel metabolite discovery. Additionally, these compounds are promising candidates for further bioactivity development.

## 1. Introduction

To date, the study of natural products has focused on the elaborate biosynthetic pathways in terrestrial plants and microorganisms. In the late 1960s, however, the search for novel metabolites took a new direction as the realm of exploration expanded to include marine plants and animals. This avenue of research was initiated primarily by academicians and facilitated by the development of scuba gear, which provided an effective means to collect shallow-water marine organisms. The study of marine natural products is recognized as both an integral component of natural product chemistry and a significant contributor to drug discovery. In addition, the success of marine natural product chemistry has helped nurture the growing discipline of marine chemical ecology, an area of research that has contributed significantly to our understanding of the ecological roles of marine secondary metabolites [1].

According to Gauthier *et al.* (1995) [2], *Alteromonas* was redefined based on phylogenetic comparisons, suggesting that the genus should be divided into two genera, *Alteromonas* (which now includes one species only) and a new genus, *Pseudoalteromonas*. This newly created genus attracted significant interest for two reasons. First, *Pseudoalteromonas* species are frequently found in association with eukaryotic hosts in the marine environment and studies of such associations will elucidate the important mechanisms in microbe-host interactions. Second, many of the species produce biologically active metabolites, which act on a range of organisms [3].

Since the identification and purification of quinine from Cinchona bark in 1820, other quinoline derivatives have been isolated from natural sources [4,5]. In particular, 2-hydroxyquinoline and 4-hydroxyquinoline, which predominantly exist as 2(1H)-quinolone and 4(1H)-quinolone, respectively, and form the core structure of several alkaloids, were isolated from plant sources. Several different animal and bacterial species also produce compounds of the quinolone class. These differ not only in terms of substitutions in the carbocyclic and hetero aromatic rings but also have other rings fused to the quinolone nucleus. Some of these naturally occurring quinolones have medicinal properties, while others have served as lead molecules in drug discovery and helped in the design of synthetic quinolones to be used as drugs. For example, *Pseudomonas aeruginosa* and related bacteria produce 2-alkyl-4(1H)-quinolones, some of which exhibit antimicrobial activity [4]. 2-Heptyl-3-hydroxy-4(1H)-quinolone, known as the *Pseudomonas* quinolone signal (PQS), belongs to the 4-quinolone family, which is best known for antimicrobial activity [6]. Furthermore, Cardozo’s group recently reported that an extracellular compound of *Pseudomonas* inhibits methicillin-resistant *Staphylococcus aureus* (MRSA) [7]. Interestingly, this naturally occurring quinolone molecule also acts as a quorum-sensing (QS) signal molecule, controlling the expression of several virulence genes as a function of cell population density [4,8].

The metabolomics approach has been recently used to classify metabolites based on metabolite-profiling studies, allowing rapid analyses of complex data and the identification of novel compounds [9,10]. The increased interest in metabolite profiling has also arisen from the potential for more comprehensive metabolite analyses using liquid chromatography-mass spectrometry (LC-MS) technology [11]. The most important advantages of LC-MS are high sensitivity and high-throughput in combination with the ability to confirm the identity of the components present in complex biological samples, as well as detection and identification of unknown or unexpected compounds [12,13,14].

In this context, we report here the isolation and identification of a marine, bacterial strain capable of producing various novel, secondary metabolites such as quinolone alkaloids and rapid-screening technology for marine-metabolite structure identification using searchable in-house MS/MS spectral library combined with high-resolution MS. We report the production of nine pseudane series including 2-isopentylqunoline-4-one and 2-(2,3-dimetylbutyl)quinoline-4-one from marine bacterium *Pseudoalteromonas* sp*.* M2 wild-type strain. In addition, we studied the anti-melanogenic activity of the main isolated compounds in Melan-A cells to detect potential whitening properties.

## 2. Results and Discussion

### 2.1. Screening and Identification of SW1-1 Strain

**Figure 1:**
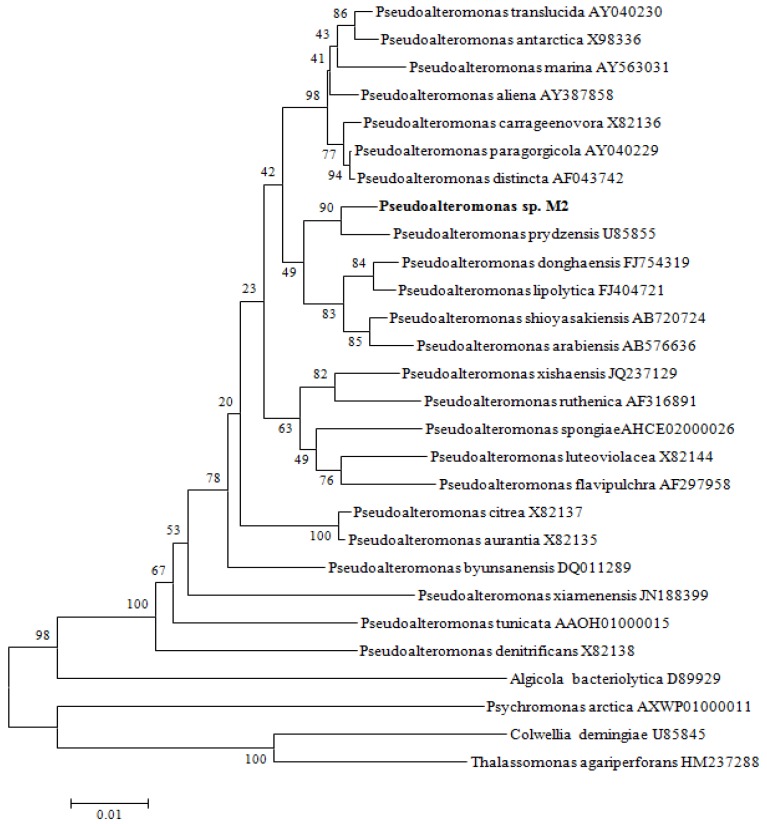
Unrooted neighbor-joining phylogenetic tree based on 16S rDNA gene sequences showing the taxonomic positions of *Pseudoalteromonas* sp. M2 and type strains of closely related taxa. The degree of confidence for each branch point was determined by bootstrap analysis (1000 replicates). Reference strains have been included in the alignment. Bar, 0.005 accumulated changes per nucleotide.

A total of 720 strains of bacteria were isolated from the intestine of the golden sea squirt. All bacterial isolates were examined for their antibacterial ability against *Vibrio anguillarum* on solid media. A few isolates showing antibacterial activity was reconfirmed in liquid culture and the one colony showing the highest antimicrobial activity, designated strain M2, was selected for further study. The complete 16S rDNA sequence (1546 bp) of strain M2 was obtained. In the phylogenetic tree constructed using unrooted neighbor-joining algorithm, strain M2 fell within the clade comprising *Pseudoalteromonas* strains (Figure 1). It exhibited 16S rDNA gene sequence similarity values between 98.69% and 98.33% to *Pseudoalteromonas prydzensis*, *P. atlantica*, and *P. espejiana*, and between 95.33% to 98.31% to type strains of other *Pseudoalteromonas* species used in the phylogenetic analysis. This sequence has been submitted to GenBank and received accession number KJ407077. These results show that M2 was a *Pseudoalteromonas* strain, designated as *Pseudoalteromonas* sp. M2.

### 2.2. Identification of Secondary Metabolites Using High-Resolution Mass Spectrometry

**Figure 2:**
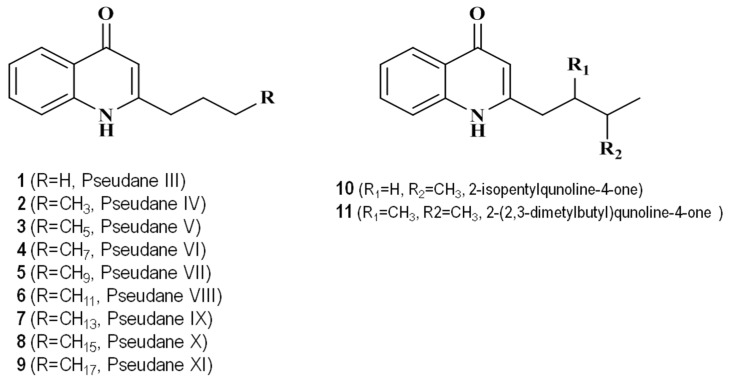
Structures of 11 4-hydroxy-2-alkylquinolines (pseudane) series compounds identified from the culture broth of *Pseudoalteromonas* sp. M2.

Table 1 shows the 4-quinolones identified in the culture broth extract from *Pseudoalteromonas* sp. M2. Analysis of the ethyl acetate extract using ultra high performance liquid chromatography with high-resolution mass spectrometry (UHPLC-HRMS) revealed 11 quinolone compounds. The structure of the secondary metabolite from *Pseudoalteromonas* sp*.* M2 culture extract is shown in Figure 2. The high-resolution mass and MS/MS spectral characteristics of the 4-quinolones were compared to commercially obtained standards and published data.

After extraction by ion chromatography (XIC), the LC-MS data were manually sorted to list such information as the retention time, *m/z* values for [M + H]^+^, and MS/MS fragmentation pattern, from base-peak chromatograms (Table 1). Each high-resolution MS spectrum and MS/MS spectrum peak were identified by AntiBase 2013 and in-house MS/MS spectral library, respectively. The differences in *m/z* values and retention time shifts were in accordance with the sequential decreases or increases in the alkyl chain length on pseudane compound. All pseudane analogues had a main common fragment ion at *m/z* 159 that was derived from the loss of the alkyl chain.

**Table 1:** Screening of secondary metabolites found in crude extracts of *Pseudoalteromonas* sp. M2.

RT (min)	Compound	[M + H]^+^ (*m/z*)	Formula	∆ppm	MS/MS Fragment Ion	Ref.
4.56	Pseudane-III	188.1070	C_12_H_14_ON	−0.003	132, 146, 159, 170, 188	
5.32	Pseudane-IV	202.1227	C_13_H_16_ON	0.491	132, 146, 159, 172, 183, 202	*
5.99	2-isopentylquinolin-4-one	216.1382	C_14_H_18_ON	−0.188	132, 146, 159, 172, 186, 200, 216	Novel
6.09	Pseudane-V	216.1382	C_14_H_18_ON	−0.188	132, 146, 159, 172, 186, 197, 216	[15]
6.71	2-(2,3-dimethylbutyl) quinolin-4-one	230.1539	C_15_H_20_ON	0.083	132, 146, 159, 172, 186, 200, 230	Novel
6.83	Pseudane-VI	230.1539	C_15_H_20_ON	−0.090	132, 146, 159, 172, 186, 200, 230	*
7.55	Pseudane-VII	244.1695	C_16_H_22_ON	−0.372	132, 146, 159, 172, 186, 200, 244	[16]
8.29	Pseudane-VIII	258.1851	C_17_H_24_ON	−0.701	132, 146, 159, 172, 186, 200, 258	*
9.00	Pseudane-IX	272.2007	C_18_H_26_ON	−0.885	132, 146, 159, 172, 186, 200, 272	[16]
9.67	Pseudane-X	286.2164	C_19_H_28_ON	−0.131	146, 159, 172, 186, 200, 214, 286	[17]
10.33	Pseudane-XI	300.2322	C_20_H_30_ON	−0.001	146, 159, 172, 186, 200, 214, 300	[17]

* Commercial source.

**Figure 3:**
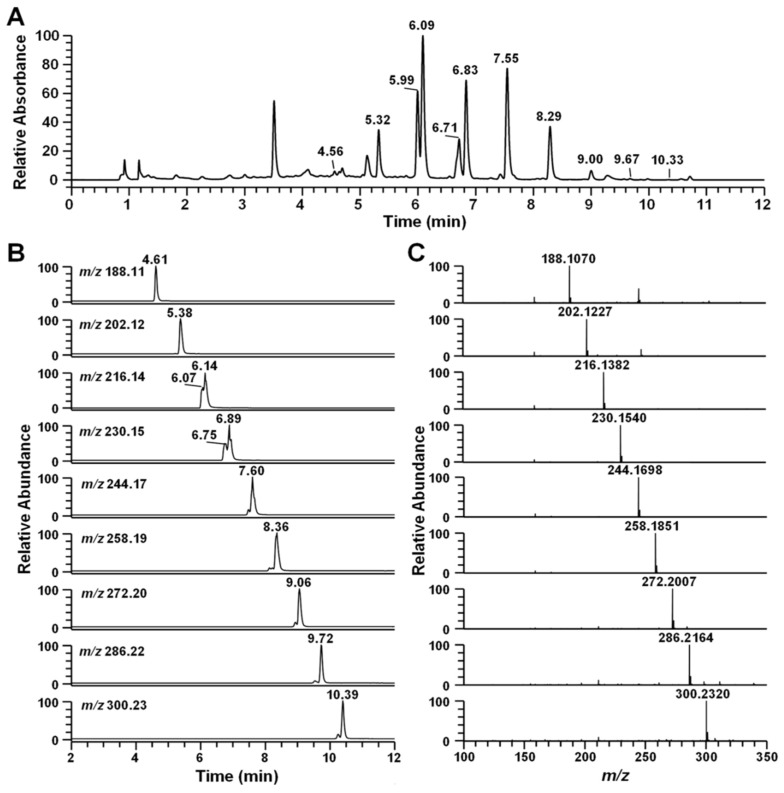
Ultra-high-pressure liquid chromatography (UHPLC)-high-resolution mass spectrophotometry (LC-HR-MS) analysis of the ethyl acetate extract from *Pseudoalteromonas* sp. M2. culture supernatant. (**A**) UHPLC chromatogram of ethyl acetate extract from *Pseudoalteromonas* sp. M2; (**B**) extracted mass chromatogram (XIC) of 11 4-quinolones. Pseudane-III (4.61 min), pseudane-IV (5.38 min), 2-isopentylquinolin-4-one (6.07 min) pseudane-V (6.14 min), 2-(2,3-dimethylbutyl)quinolin-4-one (6.75 min), pseudane-VI (6.89 min), pseudane-VII (7.60 min), pseudane-VIII (8.36 min), pseudane-IX (9.06 min), pseudane-X (9.72 min), and pseudane-XI (10.39 min); (**C**) high-resolution mass spectrum of 11 pseudane compounds. The molecular weight of 2-isopentylquinolin-4-one and pseudane-V as well as 2-(2,3-dimethylbutyl)quinolin-4-one and pseudane-VI were similar.

Searchable MS/MS spectra libraries based on the results of the liquid chromatography coupled with electrospray ionization (ESI) and tandem mass spectrometry (LC-MS/MS) with data-dependent acquisition using an ion trap mass spectrometer were compiled with regard to the identification and confirmation of the secondary metabolites from *Pseudoalteromonas* sp*.* M2. The main compound was identified as pseudane-V by AntiBase database search and confirmed by comparison analysis with a standard. The others peaks were detected before and after the major peaks (Figure 3A). The high-resolution mass spectrum showed an *m/z* 188.1070, 202.1227, 216.1382, 230.1540, 244.1698, 258.1851, 272.2007, 286.2164, and 300.2320 ([M + H]^+^), which was tentatively identified as pseudane-III to XI based on the high-resolution mass and MS/MS production ions, respectively (Figure 3C, Table 1). Thus, the LC-MS/MS analysis of the *Pseudoalteromonas* sp*.* M2 strains identified nine secondary metabolite peaks as known or putative structures, including pseudane-III (4.61 min), pseudane-IV (5.38 min), pseudane-V (6.14 min), pseudane-VI (6.89 min), pseudane-VII (7.60 min), pseudane-VIII (8.36 min), pseudane-IX (9.06 min), pseudane-X (9.72 min), and pseudane-XI (10.39 min), whereas another two peaks (6.07 and 6.75 min) were determined as unknown metabolites (Figure 3B). The regular intervals of *m/z* values and retention time-shifts of the parent ions were caused by sequential decreases or increases in the alkyl chain length.

The two unknown peaks were shown as *m/z* 216.1382 ([M + H]^+^) and *m/z* 230.1539 ([M + H]^+^), which were consistent with the molecular formula C_14_H_18_ON (∆ppm −0.188) and C_15_H_20_ON (∆ppm 0.083), respectively. Interestingly, the two unknown metabolites have the same molecular formula and very similar MS/MS pattern with pseudane-IV and pseudane-V compounds, respectively. Moreover, the unknown compounds showed the same main dissociation fragment peaks, for example, *m/z* 146 and *m/z* 159, and these fragment peaks were derived from 4-hydroxy-2-alkylquinoline backbone by the loss of an alkyl chain. According to these data, the unknown peaks were clearly shown to be a new pseudane isomer.

Previously, Lépines’s group reported 4-hydroxy-2-alkylquinolines series (pseudan-V~XIII) produced by a genetically engineered strain *pqs*L mutant derivative of PA14, indicating that this gene was involved in the biosynthesis of 4-hydroxy-2-alkylquinoline compounds in pathogenic *Pseudomonas aeruginosa* [17]. In this study, the novel *Pseudoalteromonas* sp. M2 strain producing a pseudane series with two novel compounds was screened using LC-MS based secondary metabolite screening methodology, from a total of 720 wild-type marine bacterial candidates.

### 2.3. NMR Analysis of the New Compounds

For structural analysis of secondary metabolites, 16 L of cell culture medium was centrifuged and the crude extracted using ethyl acetate. The pseudane-IV, pseudane-V, pseudane-VI, pseudane-VII, pseudane-VIII, pseudane-IX, and two unknown compunds were purified using an AutoPurification system (Waters, Milford, MA, USA; data not shown) and obtained 3.07, 14.96, 10.21, 11.37, 3.18, 1.10, 5.48, and 2.11 mg, respectively. The two unknown compounds were determined as new metabolites (2-isopentylquinolin-4-one and 2-(2,3-dimethylbutyl)quinolin-4-one) by NMR analysis (Table 2). The ^1^H NMR analysis of 2-isopentylquinolin-4-one chemical shifts (400 MHz, CD_3_OD) were: δ ppm 0.99 (3H, m, *J* = 8.3, 7.0, 1.3 Hz), 0.99 (3H, m, *J* = 8.3, 7.0, 1.3 Hz), 1.67 (1H, m, *J* = 7.3 Hz), 2.72 (2H, m, *J* = 8.5 Hz), 1.63 (2H, m), 6.22 (1H, s), 7.58 (1H, ddd, *J* = 8.46 Hz), 7.38 (1H, d, *J* = 8.16, 7.03, 1.13 Hz), 8.20 (1H, dd, *J* = 8.53, 1.26 Hz), 7.68 (1H, t, *J* = 7.39 Hz). The ^1^H NMR of 2-(2,3-dimethylbutyl)quinolin-4-one chemical shifts (400 MHz, CD_3_OD) were: δ ppm 0.86 (3H, d, *J* = 6.78 Hz), 0.95 (3H, d, *J* = 6.78 Hz), 0.97 (3H, d, *J* = 6.78 Hz), 1.69 (1H, d, *J* = 13.58, 6.82, 4.39 Hz), 2.83 (1H, dd, *J* = 13.8, 5.52 Hz), 1.89 (1H, m), 6.21 (1H, s), 7.39 (1H, t, *J* = 7.54 Hz), 7.59 (1H, d, *J* = 8.03 Hz), 8.21 (1H, d, *J* = 8.2 Hz), 7.68 (1H, m). The structure and bioactivity of these two novel compounds has not been previously reported.

**Table 2:** The NMR data of new compounds, recorded at ^1^H-400 MHz; ^13^C-100 MHz in Methanol-d_4_.

No. C/H	δC ppm	δH (ppm), Integration, Multiplicity, J (Hz)
**2-isopentylquinolin-4-one**		
1 (CH_3_)	22.8	0.99 (3H, m, *J* = 8.3, 7.0, 1.3 Hz)
2 (CH_3_)	22.8	0.99 (3H, m, *J* = 8.3, 7.0, 1.3 Hz)
5 (CH)	29.2	1.67 (1H, m, *J* = 7.3 Hz)
3 (CH_2_)	33.2	2.72 (2H, m, *J* = 8.5 Hz)
4 (CH_2_)	39.3	1.63 (2H, m)
6 (CH)	108.8	6.22 (1H, s)
7 (CH)	119.2	7.58 (1H, ddd, *J* = 8.46 Hz)
8 (CH)	125.2	7.38 (1H, d, *J* = 8.16, 7.03, 1.13 Hz)
11 (C)	125.6	
9 (CH)	126.11	8.20 (1H,dd, *J* = 8.53, 1.26 Hz)
10 (CH)	133.5	7.68 (1H, t, *J* = 7.39 Hz)
12 (C)	141.7	
13 (C)	157.5	
14 (C)	180.8	
**2-(2,3-dimethylbutyl)quinolin-4-one**		
1 (CH_3_)	15.2	0.86 (3H, d, *J* = 6.78 Hz)
2 (CH_3_)	18.2	0.95 (3H, d, *J* = 6.78 Hz)
3 (CH_3_)	20.6	0.97 (3H, d, *J* = 6.78 Hz)
5 (CH)	33.4	1.69 (1H, d, *J* = 13.58, 6.82, 4.39 Hz)
4 (CH_2_)	39.9	2.83 (1H, dd, *J* = 13.8, 5.52 Hz)
6 (CH)	40.5	1.89 (1H, m)
7 (CH)	109.8	6.21 (1H, s)
8 (CH)	119.2	7.39 (1H, t, *J* = 7.54 Hz)
9 (CH)	125.2	7.59 (1H, d, *J* = 8.03 Hz)
12 (C)	125.6	
10 (CH)	126.1	8.21 (1H, d, *J* = 8.2 Hz)
11 (CH)	133.5	7.68 (1H, m)
13 (C)	141.7	
14 (C)	156.8	
15 (C)	180.5	

### 2.4. Anti-Melanogenic Effect of Secondary Metabolites and New Compounds

**Figure 4:**
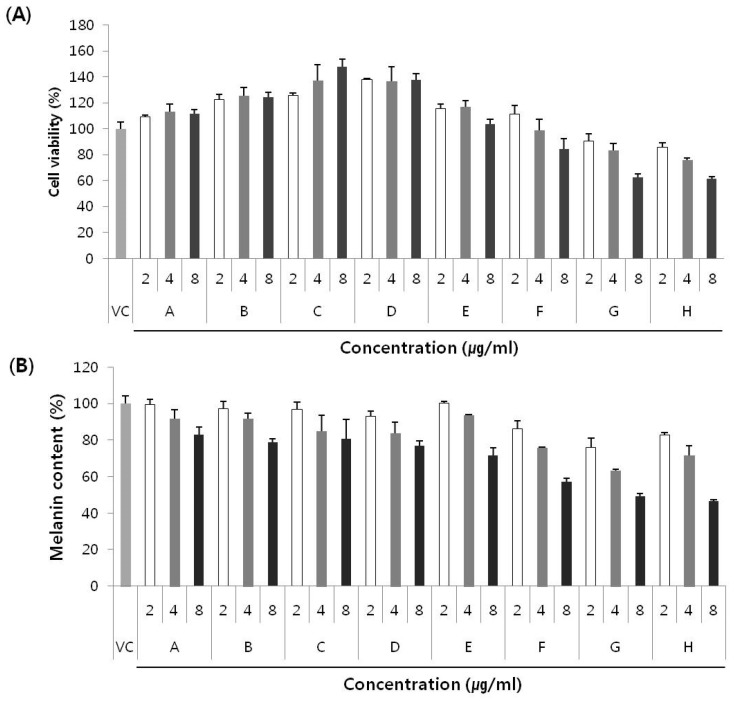
(**A**) Cell cytotoxicity and (**B**) inhibition of melanin synthesis in melan-a cells by eight main compounds produced by *Pseudoalteromonas* sp. M2. Melan-A cells were treated with compounds for 72 h and cell cytotoxicity was determined by CCK-8 cell assay. Each value is expressed as mean ± standard deviation (SD) from triplicate experiments. A: pseudane-IV, B: 2-isopentylquinolin-4-one, C: pseudane-V, D: 2-(2,3-dimethylbutyl)quinolin-4-one, E: pseudane-VI, F: pseudane-VII, G: pseudane-VIII, H: pseudane-IX.

To investigate the cytotoxic effects of the new compounds on melan-a cells, the cells were exposed to 2 to 8 µg/mL of pseudane-IV, 2-isopentylquinolin-4-one, pseudane-V, 2-(2,3-dimethylbutyl) quinolin-4-one, pseudane-VI, and pseudane-VII, pseudane-VIII, or pseudane-IX for three days, following which, cell viability was assessed using the CCK8 assay kit. None of the tested compounds exhibited toxicity at the concentration of 8 µg/mL, except for pseudane-VIII and IX (Figure 4A). Anti-melanogenic effect was measured in terms of melanin content in the presence of 2 to 8 µg/mL of the test compounds. As shown in Figure 4B, all compounds showed an inhibitory effect on melanin synthesis in a dose-dependent manner. Among the eight compounds, 2-(2,3-dimethylbutyl)quinolin-4-one, pseudane-VI, and pseudane-VII showed 23.0%, 28.2%, and 42.7% inhibition of melanin synthesis in the melan-a cells, respectively. Especially, pseudane-VII showed the highest inhibitory activity (42.7%) at a concentration of 8 µg/mL. It has been reported that few active anti-melanogenic agents such as ginsenosides extracted from leaves of *Panax ginseng* [18] showed 35.5% inhibitory activity at 80 µM. In addition, cinnamic acid extracted from *Cinnamomum cassia* Blume and *Panax ginseng* exhibited 29% inhibitory effect on melanin synthesis at 500 µM [19]. Compared with ginsenoside and cinnamic acid, pseudane-VII showed strong inhibitory activity at concentrations of 8–33 µM (the concentration unit was converted from µg/mL to µM for comparison). However, the mechanism of the anti-melanogenic activity has not yet been investigated. Therefore, further *in vitro* and *in vivo* studies are necessary to determine the mechanism involved in the anti-melanogenic effect exerted by treatment with pseudane-VII.

## 3. Experimental Section

### 3.1. Isolation of Pseudane-Producing Bacterium

Golden sea squirt (*Halocynthia aurantium*) was collected from the East Sea, South Korea, and used as a source to isolate bacteria. Strain M2 was isolated by the standard dilution plating technique using marine agar 2216 (Becton Dickinson, Franklin Lakes, NJ, USA) with 3 g/L yeast extract (Difco) and 5 g/L protease peptone (Difco) at 22 °C and incubated under routine culture conditions. Microbial strains were isolated after incubation for two or three days. All bacterial isolates were examined for their antibacterial activity against *Vibrio anguillarum* on agar media. The isolate showing the largest halo, designated M2, was studied further.

### 3.2. Identification of Pseudoalteromonas *sp.*

Genomic DNA was extracted from *Pseudoalteromonas* sp. M2 for 16SrDNA analysis as previously described [20]. Polymerase chain reaction (PCR) was performed to amplify the 16S rDNA coding region using primers 5′-AGAGTTTGATCCTGGCTCAG-3′ and 5′-ACGGTTACCTTGTTACGACTT-3′. The reaction mixture was subjected to initial denaturation at 95 °C for 10 min, followed by 30 cycles of denaturation at 95 °C for 1 min, annealing at 55 °C for 1 min, and extension at 72 °C for 1 min, with a final extension at 72 °C for 10 min, using a thermal cycler (TaKaRa, Shiga, Japan). The PCR product was subcloned into pGEM-T Easy vector, and transformed into *Escherichia coli* DH5α. DNA sequencing was performed using an Applied Biosystem Automated DNA Sequencer model 3130 with a dye-labeled terminator sequencing kit (Applied Biosystems, New York, NY, USA).

An unrooted neighbor-joining tree for the full sequence of the 16SrDNA was constructed based on the Kimura two-parameter model. Reference strains have been incorporated in the alignment and they were obtained from NCBI (http://www.ncbi.nlm.nih.gov). The sequences were aligned using Clustal X software [21], and the tree has been constructed using the MEGA 4 Software [22].

### 3.3. Secondary Metabolite-Profiling Using LC-MS

Strain M2 was inoculated into 5 mL marine broth 2216 (MB; Difco) and incubated for 48 h at 22 °C with shaking. The culture broth was transferred to a 15 mL tube and centrifuged at 11,000 *g* for 10 min. The supernatant was extracted with an equal volume of ethyl acetate. The dried ethyl acetate extract of M2 was dissolved in 50% methanol and 5 µL was analyzed to identify secondary metabolites by LC-MS technique.

All LC/MS analyses were carried out using an LTQ Orbitrap XL (Thermo Electron Co., Madison, WI, USA) coupled to an Accelar ultra-high pressure liquid chromatography system (Thermo, Waltham, MA, USA). Chromatographic separation of metabolites was conducted using a ACQUITY UPLC^®^ BEH C_18_ column (2.1 × 150 mm, 1.7 μm, Waters, Milford, MA, USA), operated at 40 °C and using mobile phases A (water) and B (acetonitrile with 0.1% formic acid) at flow rate of 0.4 mL/min. The initial gradient composition (95% A/5% B) was held for 0.5 min, increased to 80% B in 10 min, decreased to 0% A in 10.01 min, and held for 1.90 min. For recycling, the initial gradient composition was restored and allowed to equilibrate for 3 min. The LC-MS system consisted of heated electrospray ionization probe (HESI-II) as the ionization source. HESI was operated at 300 °C with spray voltage of 5.0 kV. The nebulizer sheath and auxiliary gas flow rates were set at 50 and 5 arb, respectively. MS analysis was performed with polarity switching, and the following parameters for MS/MS scan: *m*/*z* range of 100–1000; collision-induced dissociation energy of 45%; data-dependent scan mode. The Orbitrap analyzer was used for high-resolution mass spectra data acquisition with a mass resolving power of 30,000 FWHM (Full width at half maximum) at *m/z* 400. The data-dependent tandem mass spectrometry (MS/MS) experiments were controlled using menu-driven software provided with the Xcalibur system. All experiments were performed under automatic gain control conditions.

### 3.4. Extraction and Purification of Secondary Metabolites

To obtain secondary metabolites, the cell culture medium was centrifuged at 20,000 *g* for 30 min to remove precipitates. The supernatant was collected and treated with equal volume of ethyl acetate and shaken at 300 rpm for 15 min using a JEIO TECH RS-1 recipro shaker (Jeio Tech, Daejeon, Korea). Then the ethyl acetate layer (upper layer) was vacuum-dried using Speed-Vac (Labconco, Kansas, MA, USA) and the extract was diluted in 50% methanol (*v*/*v* in deionized water) to achieve concentrations of 540 mg/10 mL. Each extract was purified by high-pressure liquid chromatography (HPLC) on a Waters AutoPurification System (Waters, Milford, MA, USA) with a QDa detector and a Waters Xbridge prep C_18_ Column (19 × 250 mm, 5 μm) with a gradient of A (0.1% formic acid *v*/*v* in deionized water) and B (acetonitrile) at flow rate of 25 mL/min. The initial gradient composition (90% A/10% B) was held for 2.8 min, increased to 65% B in 43 min, and then decreased to 0% A in 45 min, where it was held for 5 min.

### 3.5. Nuclear Magnetic Resonance (NMR) Analysis

^1^H and ^13^C NMR spectra were recorded on Bruker Avance II 400 (Bruker, Billerica, MA, USA) in MeOD solutions. Working frequencies were 400.1 and 101.0 MHz for ^1^H and for ^13^C, respectively.

### 3.6. Cell Cultures

The Melan-A (murine Melan-A melanocyte) cell line, originally derived from C57BL/6 J (black, a/a) mice was received as a gift from Prof. Dorothy C. Bennett (St George’s Hospital Medical School, London, UK). Melan-A cells are similar in characteristics to melanocytes *in vivo* and are widely used as a suitable substitute for normal primary mouse melanocytes in melanin metabolism tests. This cell line was cultured in RPMI 1640 supplemented with 10% fetal bovine serum (FBS), streptomycin-penicillin (100 µg/mL each), and 200 nM 12-*O*-tetradecanoylphorbol-13-acetate (TPA), a potent tumor promoter, at 37 °C in 5% CO_2_. Cells were subcultured every three days up to a maximum of 40 passages. Confluent monolayers of melanocytes were harvested with a mixture of 0.05% trypsin and 0.53 mM EDTA (Gibco BRL, Grand Island, NY, USA).

### 3.7. Cell Viability Assay

Cell viability was determined via crystal violet staining. After four days of incubation with the test substances, the culture medium was removed and replaced with 0.1% crystal violet in 10% ethanol. The cells were then stained for 5 min at room temperature and rinsed with phosphate-buffered saline (PBS) three times. The crystal violet stain retained by adherent cells was extracted using 95% ethanol and absorbance was determined at a wavelength of 590 nm.

### 3.8. Measurement of Melanin Content

The cells were seeded in a 24-well plate (Corning, NY, USA) at a density of 1 × 10^5^ cells per well and allowed to attach overnight. They were then incubated in a fresh medium containing various concentrations of the test compounds for four days. After the cells had been washed with PBS, they were lysed with 250 μL 0.85 N KOH and transferred to a 96-well plate. The melanin content was estimated via absorbance measurements at a wavelength of 405 nm.

## 4. Conclusions

*Pseudoalteromonas* sp*.* M2 isolated from marine source was found to produce various secondary metabolites and novel compounds. Based on high-resolution MS and NMR spectroscopic analysis, two novel compounds, 2-isopentylquinolin-4-one and 2-(2,3-dimethylbutyl)quinolin-4-one are identified. The production of 9 quinolones (pseudane series III–XI), 2-isopentylqunoline-4-one, and 2-(2,3-dimetylbutyl)qunoline-4-one from a single wild-type marine bacterium has not been previously reported. We confirmed biological activity of the isolated compounds, including inhibition of melanin synthesis in Melan-A cells. This may be a useful approach to evaluate multi-functional biological activities to explore the potential therapeutic applications of this bacterium. Pseudane-VI, VII, and 2-(2,3-dimethylbutyl)quinolin-4-one may be promising candidates for the development of useful skin-lightening agents.

As shown in the present study, LC-MS/MS-based metabolite profiling of *Pseudoalteromonas* sp*.* M2 secondary metabolites is a useful technique to distinguish between known and unknown compounds, as well as to screen novel compounds without extensive culturing. Furthermore, the structure-based metabolite screening method, high-resolution LC-MS combined with MS/MS spectral library searches, minimizes both, time and resources utilized in redundant discovery efforts. The objective of this study was to develop a rapid, accurate, and more efficient technique compared with traditional biological activity-based screening methods for discovery of novel compounds from natural sources.
